# The Prevalence and Characteristics of Breakthrough Cancer Pain in Patients Receiving Low Doses of Opioids for Background Pain

**DOI:** 10.3390/cancers13051058

**Published:** 2021-03-02

**Authors:** Sebastiano Mercadante, Marco Maltoni, Domenico Russo, Claudio Adile, Patrizia Ferrera, Romina Rossi, Marta Rosati, Alessandra Casuccio

**Affiliations:** 1 Main Regional Center for Pain Relief and Supportive/Palliative Care, La Maddalena Cancer Center, 90146 Palermo, Italy; claudio.adile@hotmail.it (C.A.); patriziaferrera@inwind.it (P.F.); 2 Palliative Care Unit, Istituto Scientifico Romagnolo per lo Studio e la Cura dei Tumori (IRST) IRCCS, 47014 Meldola, Italy; marcocesare.maltoni@auslromagna.it (M.M.); romina.rossi@irst.emr.it (R.R.); marta.rosati@auslromagna.it (M.R.); 3 Hospice and the Palliative Care Service, Clinica San Marco, 04100 Latina, Italy; dt.domenico.russo@tiscali.it; 4 Department of Health Promotion, Mother and Child Care, Internal Medicine and Medical Specialties (PROMISE), University of Palermo, 90133 Palermo, Italy; casuccio@unipa.it

**Keywords:** breakthrough cancer pain, opioids, doses

## Abstract

**Simple Summary:**

The aim of this study was to assess the prevalence and characteristics of breakthrough cancer pain (BTcP) in patients receiving low doses of opioids for background pain. This prospective study showed that in this population, BTcP prevalence was 69.8%. Many patients did not achieve a sufficient level of satisfaction with BTcP medications, particularly with oral morphine. Data also suggest that better optimization of background analgesia, though apparently acceptable, may limit the number of BTcP episodes.

**Abstract:**

The aim of this study was to assess the prevalence and characteristics of breakthrough cancer pain (BTcP) in patients receiving low doses of opioids for background pain. A consecutive sample of advanced cancer patients receiving less than 60 mg/day of oral morphine equivalent (OME) was selected. Epidemiological data, background pain intensity, and current analgesic therapy were recorded. The presence of BTcP was diagnosed according to a standard algorithm. The number of BTcP episodes, intensity of BTcP, its predictability and triggers, onset duration, interference with daily activities, BTcP medications, satisfaction with BTcP medication, and time to meaningful pain relief were collected. A total of 126 patients were screened. The mean intensity of background pain was 2.71 (1.57), and the mean OME was 28.5 mg/day (SD15.8). BTP episodes were recorded in 88 patients (69.8%). The mean number/day of BTP episodes was 4.1 (SD 7.1, range 1–30). In a significant percentage of patients, BTcP was both predictable and unpredictable (23%). The BTcP onset was less than 20 min in the majority of patients. The mean duration of untreated episodes was 47.5 (SD 47.6) minutes. The mean time to meaningful pain relief after taking a BTcP medication was >20 min in 44.5% of patients. The efficacy of BTcP medication was not considered good in more than 63% of patients. Gender (females) (OR = 4.16) and lower Karnofsky (OR = 0.92) were independently associated with BTcP. A higher number of BTcP episodes/day was associated with gender (females) (*p* = 0.036), short duration of BTcP (*p* = 0.005), poorer efficacy of BTcP medication (none or mild) (*p* = 0.001), and late meaningful pain relief (*p* = 0.024). The poor efficacy of BTcP medication was independently associated with a higher number of episodes/day (OR = 0.22). In patients who were receiving low doses of opioids, BTcP prevalence was 69.8%. Many patients did not achieve a sufficient level of satisfaction with BTcP medications, particularly with oral morphine. Data also suggest that better optimization of background analgesia, though apparently acceptable, may limit the number of BTcP episodes.

## 1. Introduction

Pain is one of the most common and feared symptoms of cancer, with a prevalence largely differing according to the stage of the disease [1]. Other than background pain, a variable percentage of cancer patients may develop a transitory peak of pain intensity, commonly understood as breakthrough pain (BTcP). Identification of BTcP depends on the parameters selected for its definition [2,3,4,5].

At present, BTcP is understood to be an episode of severe pain, of variable duration, that occurs in patients with chronic pain who are receiving a stable opioid-based analgesic regimen and who describe their baseline pain as both stable and mild to moderate in intensity for most hours of the day [6,7]. The detection and measurement or BTcP may be enhanced by narrowing the criteria for the definition, thus increasing the reproducibility of assessments. However, a narrower definition may result in the exclusion of clinically important episodic pains from consideration. Specific criteria used to identify BTcP have attempted to achieve a balance between the value of precision and the risk of oversimplifying a complex phenomenon [4,8,9,10,11,12]. Some criteria appear to be largely shared in literature. First, to identify BTcP, the patient must be receiving a stable opioid regimen, and baseline pain must be controlled over some period of time [13,14,15]. Second, the presence of a precipitating event should be used to describe the subtype of BTcP as spontaneous or incident-type [6], and not to exclusively designate a BTcP. Spontaneous BTcP, labeled as idiopathic BTcP, lacks an identifiable cause or precipitating event. Its onset and duration are often relatively longer than in incident-type BTcP, triggered by an identifiable event. The most common type of incident BTcP is movement-induced bone pain due to metastatic disease and swallow-induced oropharyngeal pain from mucositis.

The principal characteristic of BTcP episodes is the temporal pattern of a short onset and offset, that should be clearly distinguished from the background pain. In pioneer surveys of BTcP, a set of characteristics for the purpose of identifying the phenomenon [6,7] has been provided by researchers to offer more insights on BTcP onset, duration, predictability, and triggering factors, number of episodes/day, background pain intensity, medications used for background pain and for BTcP, time to achieve meaningful analgesia after BTcP medications, and choice of an effective BTcP medication with minimal adverse effects have been extensively assessed in these last years.

The principal finding of studies published in the last decade was that BTcP is a variegate phenomenon, often presenting in different ways in individuals that may change its presentation during the course of the patient’s disease in the same individual [7,16,17,18], as would be a chameleon [19]. Accordingly, the prevalence of this phenomenon has been reported to be variable in the literature, due to the different settings, assessment methods, stage of disease, level of basal analgesia, medications, and doses used for background analgesia, and characteristics of BTcP [4,5]. For example, BTcP can develop in different conditions: in highly opioid-tolerant patients, that means receiving high doses of opioids for background analgesia, in patients receiving low doses of opioids (in the range of so-called weak opioids, that is less than 60 mg/day of oral morphine equivalents (OME)), or even in patients not receiving opioids [20]. It is evident that any condition may have its own peculiarity that may reflect different characteristics, consequently requiring different treatment strategies, either in terms of drugs or dosages.

Indeed, there is typically no distinction made between BTcP that can be clearly attributed to an etiology associated with increased pain and BTcP that could be related to insufficient opioid therapy, as long as the stable dose of the opioid is associated with controlled baseline pain. In many epidemiological studies of BTcP, patients receiving non-opioids or opioids for moderate pain were indistinctly included [4,8,9,10,11,12,21]. A recent study has shown that patients receiving lower doses of opioids exhibit some differences in BTcP presentation in comparison with patients receiving ≥60 mg/day of OME. This group of patients reported less episodes of BTcP, with a faster onset, a lower intensity, a longer time to meaningful pain relief, and less satisfaction with BTcP medications [22]. However, this secondary analysis was performed in patients with a diagnosis of BTcP. Thus, the prevalence of this phenomenon in this subpopulation remains unknown. The aim of this prospective multicentre study was to assess the prevalence and characteristics of BTcP in patients receiving low doses of opioids for their background pain. The secondary aim was to assess the possible factors associated with BTcP and response to BTcP medications.

## 2. Methods

### 2.1. Study Design

This was a prospective, cross-sectional, multicenter study performed during a period of one year in three palliative care centers (Palermo, Forlì’, and Latina), from January to December 2019. Patients were seen as outpatients (*n* = 25) or inpatients (hospice or palliative care unit, *n* = 101). The study was approved by the ethical committee of the University of Palermo (no. 06/2018, 11 July 2018), and written informed consent was obtained from each patient.

Inclusion criteria were age ≥18 years, a diagnosis of advanced cancer, the use of opioids in doses of less than 60 mg/day of OME for the management of background cancer pain, and a background pain considered to be acceptable by patients, not requiring changes in opioid doses given around the clock. Exclusion criteria were poor collaboration, a short life expectancy (less than 2 weeks), and unstable or uncontrolled background pain (>4/10).

Data were collected by experienced palliative care physicians. Age, gender, primary cancer, stage of disease, and Karnofsky status were recorded. The following data were collected: pain mechanisms (nociceptive (somatic-visceral), neuropathic, or mixed), average pain intensity in the last 24 h (on a numerical scale of 0–10), opioids used for background pain and their doses, expressed as OME, and the presence of BTcP. The diagnosis of BTcP was based on a previously reported algorithm [12]: a peak of moderate-severe intensity, well-distinguished from background pain acceptable for patients and not requiring changes in opioid doses (generally, pain intensity of ≤4 on a 0–10 numerical scale). The characteristics of BTcP were examined: number of episodes/day, intensity, onset (5–10 min, 11–15 min, 16–20 min, >20 min), duration of an untreated episode, predictability and triggering factors, medications and doses used for BTcP, and mean time to meaningful pain relief after taking a BTcP medication (<10 min, 10–20 min, 21–30 min, >30 min). Patients were asked about the efficacy of BTcP medication (none, mild, good, very good). BTcP medications and their doses were prescribed according to local policy and choices by experienced physicians.

### 2.2. Statistical Analysis

Categorical variables were described as absolute (*n*) and relative (%) frequencies; mean and standard deviation (SD) summarized continuous variables. To compare patients with or without BTcP, bivariate analyses were conducted. The differences between clinical characteristics of patients with or without BTcP were assessed by the chi-square test or Fisher exact test, as needed for categorical variables, and by the independent Student *t* test for continuous parameters if the data were normally distributed. All variables that were found to be significantly associated with the presence of BTcP (*p* ≤ 0.05) at the bivariate analysis were included in a multivariate regression model in order to avoid any potential confounding effects. We also evaluated, using a bivariate analysis, the factors associated with BTcP by categorizing the patients into two groups (with <4 and ≥4 episodes). Again, the factors found to be significantly associated were evaluated by a multivariate regression analysis. Data were analyzed by the IBM SPSS Software 22 version (IBM Corp., Armonk, NY, USA). All *p*-values were two-sided, and *p* < 0.05 was considered statistically significant.

## 3. Results

A total of 126 patients were screened according to inclusion/exclusion criteria. The general characteristics of these patients are described in Table 1.

### 3.1. Background Pain

Pain mechanisms were somatic (*n* = 35), visceral (*n* = 28), neuropathic (*n* = 6), or mixed (*n* = 54). In three patients, data were missed. The mean intensity of background pain was 2.71 (SD 1.57). Opioids used for background pain management are listed in Table 2. The mean opioid doses, expressed as OME, were 28.5 mg/day (SD 15.8).

### 3.2. Characteristics of BTcP

BTcP episodes were reported by 88 of patients (69.8%). The mean intensity of background pain in these patients was 2.6 (SD 1.5), and no differences in background pain intensity with patients without BTcP were found (*p* = 0.144). The mean doses of opioids (OME) used for background analgesia in patients with BTcP were 28.3 mg/day (SD 17.6). No differences in OME in patients with or without BTcP were found (*p* = 0.814).

The mean number of BTcP episodes was 4.17/day (SD 7.1, range 1–30). BTcP was euther predictable, unpredictable, both predictable and unpredictable, or missed in 28.4%, 47.3%, 23%, and 1.3% of patients, respectively. The main triggers were in a rank order: movement (56.8%), feeding (13.6%), urination (4.5%), hygienic procedures (2.4%), and others (22.7%). The BTcP onset was 5–10, 11–15, 16–20, and >20 min in 49.2%, 16.9%, 20.1%, and 13.8% of patients, respectively. The mean duration of untreated episodes was 47.5 (SD 47.6) min.

### 3.3. Medications for BTcP

Drugs and doses used for BTcP are reported in Table 3. Most patients were prescribed oral morphine for BTcP episodes. The mean time to meaningful pain relief after taking a BTcP medication was <10 min, 10–20 min, and 21–30 min in 9.3%, 46.2%, and 16.7% of patients, respectively. In 27.8% of patients, it was >30 min. The efficacy of BTcP medication was considered none, mild, good, and very good in 18.1%, 45.7%, 30.6%, and 5.6% of patients, respectively.

### 3.4. Factors Associated with BTcP

Gender (females, *p* = 0.034), age (*p* = 0.025), lower Karnofsky (*p* < 0.0005), and some primary tumors (breast, pancreas, liver, and gastrointestinal, *p* = 0.008) were associated with the probability to have BTcP. At multivariate analysis, gender (females) (OR = 4.16 95%CI 1.5–11.9; *p* = 0.008) and lower Karnofsky (OR = 0.92, 95%CI 0.89–0.96; *p* < 0.0005) were independently associated with BTcP.

More than four episodes/day were more likely in females (*p* = 0.036), as well as short duration of BTcP (*p* = 0.005), poorer efficacy of BTcP medication (none or mild) (*p* = 0.001), and late meaningful pain relief (*p* = 0.024). At the multivariate analysis, the poor efficacy of BTcP medication was independently associated with more than four episodes/day (OR = 0.22, 95% CI 0.1–0.7; *p* = 0.01).

## 4. Discussion

This study provided the first data regarding the prevalence of BTcP in cancer patients receiving low doses of opioids for their background pain. Females and patients with lower Karnofsky were more likely to have BTcP. This information is unexpected, particularly for the gender, as no study has properly assessed this aspect.

In studies performed in a non-selected population of cancer patients, the prevalence of BTcP has been reported to be less than 40%. This group of patients included those receiving (or not receiving) weak or strong opioids, those who had or did not have stable, well-controlled background pain, and those with different levels of BTP intensity [4,8,9,10,11,12,21]. The large variability also depended on the setting, stage of disease, background opioid analgesia, and methodology used for the diagnosis of BTP, other than the design of the studies, which was often retrospective [16,19]. Indeed, BTcP is differently observed in 50–90% of all hospitalized cancer patients, in 89% of patients admitted to homes and hospices, and in 35% of outpatients or in 83% of patients attending their first visit [23,24,25]. In a recent multicenter study performed in Spain using a specific diagnostic algorithm, BTcP prevalence in cancer patients was 48% [25]. Thus, BTcP prevalence can be different according to the way it is studied. Moreover, in many studies, no specific definition has been adopted a priori, taking together patients with poor background pain control, with no intense peaks well-distinguished from background pain, receiving or not receiving analgesics, with different doses, and able or unable to provide acceptable background analgesia [26].

Specifically, no study has ever assessed the prevalence of BTcP in patients receiving low doses of OME that is less than the traditional 60 mg/day, which is commonly considered the cut-off point of the third step of the WHO analgesic ladder. Although the distinction between weak and strong opioids seems to be obsolete, as strong opioids can be used early at low doses, this is considered the minimal dose requested to prescribe fentanyl products for BTcP. This category of drugs commonly used for BTP has been shown to be superior in onset and efficacy in comparison with placebo and oral opioids [27]. In this study, a specific algorithm, used in many previous studies and substantially shared in the literature [3], has been used to label patients with BTcP [2,12,18].

Other features regarding the characteristics of BTcP presentation have been taken into consideration. In this study of patients receiving low doses of opioids for background pain, there were not only patients with predictable or unpredictable pain, but also patients presenting both conditions. This aspect has been poorly described in the general population and deserves further study. Potentially, this subgroup of patients may have more therapeutic options, rendering the treatment more complex. Indeed, more than half of the patients were reported to experience more than one type of BTcP, although it is unclear what kind of different BTcP, with 4.3% of patients presenting with four or five different types of BTcP [14].

BTcP onset and duration did not present relevant differences in comparison with the general population [6], while a greater number of episodes was recorded in this study. Interesting associations, which were never reported before, were found. In females and patients with a lower Karnofsky, BTcP was more frequent. The finding that patients with a lower Karnofsky were more likely to have more episodes of BTcP was previously reported and could be explained with a more advanced stage of disease and metastases, able to produce some kinds of BTcP. The role of gender should be better clarified. This finding could be due to the low number of patients, the higher number of females among the recruited patients, or the prevalence of some type of cancer, namely breast cancer. Thus, this aspect, at least in this subgroup of patients receiving low doses of opioids, deserves further interest and specific study.

Moreover, having a higher number of episodes was associated with limited efficacy of BTcP medications. As a consequence, patients receiving low OMEs could also have a suboptimal management of BTcP, as reported in recent secondary analysis of a large study [22]. One can argue that these patients could be undermedicated, although they reported to apparently have an acceptable analgesia. This poses the question whether patients would have their pain optimized by a stronger background opioid analgesia. This concept could be reinforced by the finding that transmucosal fentanyl products were largely used successfully with no risks of adverse effects, despite their traditional indication to be prescribed in patients tolerant to at least 60 mg/day of OMEs, as the patient would be deserving higher doses of OME for background analgesia. The finding of a higher number of episodes per day seems to confirm such a hypothesis.

In this study, patients receiving lower OMEs were less likely to be prescribed a BTcP medication in comparison with patients receiving higher doses, and about 30% of patients of them were prescribed oral morphine [18]. In the present study, oral morphine was more frequently used (about 50%), although the mean doses of opioids were quite similar between the studies. In terms of mean meaningful time for pain relief after a BTcP medication, a significant number of patients reported to need more than 30 min to appreciate a significant change. Efficacy of BTcP medications was found to be none or mild in more than 63% of patients, which is a higher percentage compared to data reported in a similar population of patients on low doses of opioids (23%) [22]. Intuitively, these findings can be explained by the different pattern of opioids used for BTcP in the two studies (oral morphine and transmucosal fentanyl products). In a recent double-blind study comparing different doses of oral morphine with placebo, oral morphine produced minimal analgesia [28].

There are some limitations in this study. The number of patients was not as high as in other recent studies [11] and was performed in only three centers. However, this is a subgroup of patients who are not so easy to recruit in palliative care services, as most patients on low doses of opioids often still receive active treatment and are followed by oncologists. Moreover, this was a cross-sectional study. The advantages were the prospective and primary analysis of patients, performed in cooperative and homogenous research groups working together for years, and the use of a clear definition of BTcP to assess the prevalence of this phenomenon. For instance, low doses of transmucosal fentanyl products have been found to be effective and safe in patients receiving low OMEs [29]. Finally, the heterogeneity of background medication may be a further drawback of this study.

## 5. Conclusions

In conclusion, about 70% of patients receiving low doses of OME experienced BTcP episodes. This subgroup of patients is worthwhile of specific attention, as they could often receive suboptimal management of BTcP. Further studies should be performed to better assess the risk factors for BTcP, for example in females, and to find an explanation for that.

## Figures and Tables

**Table 1:** Characteristics of patients: Frequencies and Mean (SD).

Data	Mean (SD)
Age, years	66 (SD 14)
Gender M/F	55/71
Mean Karnofsky	49 (SD18)
Primary tumor:	
Breast	*n* = 27
Lung	*n* = 21
Gastrointestinal	*n* = 12
Liver	*n* = 11
Kidney	*n* = 11
Myeloma	*n* = 10
Others	*n* = 34
Mean background pain intensity	2.71 (SD 1.57)
Mean background OME (mg/day)	28.5 (SD 15.8)
Patients with BTcP	88 (69.8%)

BTcP: Breakthrough cancer pain; F: female; M: male; OME: oral morphine equivalents.

**Table 2:** Number of patients using opioid drugs used for background pain.

Opioids for Background Pain	*n*
oxycodone-naloxone	28
transdermal fentanyl	25
tapentadol	22
codeine-paracetamol	10
morphine	8
oxycodone and codeine	8
tramadol	7
oxycodone	6
hydroxymorphone	5
transdermal buprenorphine	4
oxycodone-paracetamol	2
methadone	1

**Table 3:** Drugs and doses used for Breakthrough cancer pain (BtcP).

Drugs	Percentage	Doses
oral morphine	49.3%	6.2 mg (SD 2.7)
sublingual fentanyl	13.4%	76.9 μg (SD 15.9)
paracetamol	8%	1000 mg
codeine-paracetamol	5.3%	30/325 mg
parenteral morphine	5.3%	1.5 mg (SD 3.3)
fentanyl buccal tablet	4%	100 μg
tramadol	4%	30 (SD 17.3)
others	10.7%	-

## Data Availability

The data presented in this study are available on request from the corresponding author.
